# Oral Health and Dental Care Access Among Refugees in Syracuse, NY

**DOI:** 10.5334/aogh.4739

**Published:** 2025-11-03

**Authors:** Moe Kuroda, Meghan Lewis, Nidaa Aljabbarin, Nia S. Brown, Madison P. Searles, Sistu KC, Andrea V. Shaw, Christina D. Campagna

**Affiliations:** 1 Global Health Institute, SUNY Upstate Medical University, Syracuse, New York, United States of America; 2 Department of General Medicine, Toyama University Hospital, Toyama, Japan; 3 SUNY Upstate Medical University Hospital, Syracuse, New York, United States of America; 4 School of Medicine, St. George’s University, West Indies, Grenada; 5 Norton College of Medicine, SUNY Upstate Medical University, Syracuse, New York, United States of America; 6 Department of Pediatrics, SUNY Upstate Medical University, Syracuse, New York, United States of America; 7 Department of Internal Medicine, SUNY Upstate Medical University, Syracuse, New York, United States of America; 8 Department of Public Health & Preventive Medicine, SUNY Upstate Medical University, Syracuse, New York, United States of America

**Keywords:** oral health, ental care access, efugee health, ocial determinants of health

## Abstract

*Background:* Oral health and dental care access are disproportionately limited among newly resettled refugees in the United States (US).

*Objectives:* This study aimed to describe self-reported oral health practices and dental care access among refugees in Syracuse, New York.

*Methods:* A cross-sectional survey was conducted from April to September 2023 with 60 heads of household representing 313 family members from Afghanistan, Somalia, Congo, Syria, Burma, and Nepal. Descriptive and bivariate analyses were performed.

*Findings:* Half of the participants were women (51.8%), and 29.4% were aged 10–<20 years. Over half had lived in the US for less than 5 years (51.7%). Language, difficulty finding a dentist, and lack of insurance were key barriers to dental care. Only 24.0% had access to dental care in their home country, compared to 48.5% in the US. Significant associations were found between having a dentist in the US and the last visit to the dentist (*p* < 0.05), and between years in the US and dental care access (*p* < 0.001).

*Conclusions:* Our study found that dental care access among refugees in Syracuse was limited, with disparities by country of origin and length of US residence. These findings highlight the need for targeted interventions to improve dental care access and health literacy among refugee populations in the US.

## 1. Introduction

Oral health is disproportionately affected by social determinants of health in the United States (US) [1]. One of the most vulnerable groups for oral care is the refugee population [2]. The US is one of the largest host countries that has welcomed refugees, resettling more than three million people since 1975 [3]. Even after resettlement in the US, studies have shown that multiple obstacles hinder dental care access, compounding poor oral hygiene among the refugee population at baseline [4–6].

Refugees are at high risk of poor oral conditions before and after resettlement due to a variety of reasons. Before resettlement, refugees lack access to dental care services and proper dental care facilities in their country of origin [7]. Long stays in refugee camps, where refugees also face challenges in accessing healthcare and are exposed to cariogenic foods, also impede good dental practices and care [7, 8]. After resettlement, refugees continue to face challenges in oral health. English proficiency, limitations of health insurance coverage, and acculturation, including lifestyle changes, have been identified as significant barriers to dental care access and poor oral hygiene in host countries [2, 7–11]. In one study focusing on a student-run free refugee clinic in Texas, dental issues were reported to be the most prevalent complaint [12].

Studies focusing on dental care and access for refugees in the US are limited [2, 13]. A literature review revealed one 2015 study focusing on dental utilization among newly resettled adults enrolled in Medicaid [14], and another targeting refugee children [15]. However, most other studies have focused on non-US host countries, the United Kingdom [16], Australia [17], and European countries [18], or are published review articles [5, 6, 8, 19]. There is limited updated evidence on access to dental care and oral health status among refugees hailing from multiple countries of origin and living in the US. This is an important consideration given that studies have found differences in access and dental outcomes depending on refugees’ regional and cultural backgrounds. Therefore, it is important to understand access, practices, and barriers across culturally diverse refugee groups resettled to the same location in the US.

The City of Syracuse is located within Onondaga County in Upstate New York (NY). Syracuse has welcomed over 10,000 refugees from 24 countries in the past ten years [20, 21]. According to the New York State Office of Temporary and Disability Assistance, counties in Upstate NY, including Onondaga County, resettled 95% of all refugees and special immigrant visa holders resettled to New York State (NYS) in 2024 [22]. Based on our experiences working closely with refugee care teams in Syracuse, we continue to see that refugees face substantial barriers to oral health care even after resettlement. We therefore hypothesized that refugees in Syracuse, NY, encounter significant challenges in accessing dental care, with these barriers influenced by sociodemographic and health literacy factors. The present descriptive study aimed (1) to investigate current dental care access, (2) to understand oral health literacy and practices, and (3) to determine barriers to receiving dental care among refugees in Syracuse, NY.

## 2. Materials and Methods

### 2.1 Study setting

This study was conducted in Syracuse, NY. NY has been ranked the third highest state in welcoming refugees, following Texas and California [23]. In 2024, 30% of refugees welcomed by NYS were resettled in Onondaga County, where Syracuse is located [24]. Refugees resettled in Syracuse hail from many countries, including Afghanistan, the Democratic Republic of the Congo (DRC), and Syria [22]. The State University of New York (SUNY) Upstate Medical University in Syracuse has an established Community-Clinic Partnership (CCP) with Catholic Charities Refugee Resettlement Services (CYO), a local non-governmental refugee resettlement agency [25]. This study was conducted as one of CCP’s initiatives to explore dental care access, needs, and barriers among refugees in Syracuse, NY.

### 2.2 Participant recruitment

Sixty heads of household were recruited through snowball sampling by Community Health Workers (CHWs) employed by the local refugee resettlement agency (CYO). Many CHWs are former refugees themselves and are employed with the mission of supporting refugees in their resettlement after their formal case management has ended. CHWs guide refugees with culturally sensitive communication and establish trusted relationships. The trust that resettled refugee families have in CHWs is an integral part of CCP research initiatives, as refugee families are more comfortable with CHWs administering surveys and thus help to obtain reliable answers [26]. For the present study, CHWs who were already involved in ongoing case management recruited and interviewed heads of household with whom they had established connections.

Approximately ten participants from each of the six major countries of origin represented in Syracuse were targeted for recruitment. These countries included Arabic-speaking countries, Somalia, Nepal, DRC, Afghanistan, and Burma. These countries were targeted based on discussions with the study team and CHWs, balancing the diversity of refugees represented in the Syracuse population and the practical constraints of CHWs’ workload and time. As the study was primarily descriptive rather than hypothesis-driven, the sample size was not based on a formal power calculation but on feasibility considerations, consistent with recommended approaches for exploratory community-based research [27]. According to official Onondaga County resettlement data [22], the largest refugee groups resettled in Syracuse during the past decade include those from Afghanistan, the Democratic Republic of the Congo, Syria, Burma, and Somalia. In addition, although not separately listed in county-level statistics, prior reports have documented that Bhutanese–Nepali refugees constitute one of the largest refugee communities in Syracuse [28]. Taken together, these six groups capture the predominant refugee populations in the area, supporting the representativeness of our sample.

### 2.3 Data collection

Data were collected from April 24 to September 20, 2023. CHWs recruited families and interpreted the surveys in person or over the phone. CHWs captured the survey data in an online survey form created in Research Electronic Data Capture (REDCap), a secure, web-based software platform designed to support data capture for research studies [29, 30]. Respondents (refugees) and interpreters (CHWs) received $25 gift cards as an incentive. The survey is provided in the supplementary data.

Surveys were anonymous, with no identifiable health or private information collected. The questionnaire was developed by the CCP research team in collaboration with CYO and CHWs. The survey questions were not derived from a previously validated instrument; rather, they were created through repeated discussions with refugee care teams and outreach workers to ensure cultural relevance. While not formally validated in the multiple languages used, the survey was administered by bilingual CHWs who provided real-time interpretation and clarification to maximize linguistic and cultural appropriateness. The survey included 24 items broken into three sections on demographics, family information, and perspectives on dental care. The categories asked questions about experiences of dental care in both home countries and the US, as well as self-reported oral health and dental care practices. Heads of household responded to questions in sections 1 and 3, and the full dataset was created by separating each household and creating a single data entry for each family member. The head of household was always recorded as “Family Member 1.” As a result, sixty household produced 313 family members. Children who were born in the US were excluded and marked as “missing” from certain components of data analysis, specifically questions, such as “Does your family member # have a dentist at home country?” and “Who did your family member # go to when he/she has a dental problem in your home country?” See the supplementary data for more details.

### 2.4 Data analysis

Demographics and descriptive analysis of all questions were examined with descriptive and bivariate analyses. A comparison of dental care access between the country of origin and the US was analyzed using the Chi-square test. Two-tailed tests were performed at a significance level of alpha = 0.05, and all analyses were performed using IBM SPSS Statistics version 29 (Armonk, NY). Cases and records with missing data were excluded from the bivariate analysis. Multivariate analysis was unavailable due to the small sample size for heads of household and low response rate for some questions.

#### 2.4.1 Bivariate analysis among heads of household

Bivariate analysis was conducted using Chi-square and Fisher’s exact tests to explore the relationships between demographics, dental healthcare, and health insurance variables among heads of household. Demographic variables included country of origin (Afghanistan, Burma, DRC, Nepal, Somalia, and Syria). Years in the US were continuous and categorized as (<1 year, 1–<5 years, 5–<10 years, and 10+ years). Dental healthcare variables included the presence of a dentist in home countries (Yes, No, Not sure, Not applicable), the presence of a dentist in the US (Yes, No, Not sure, Not applicable), and where the head of household seeks dental care in the US (dentist, primary care physician, urgent care, emergent care, other). Health insurance variables include knowledge of clinics accepting dental insurance (Yes, No, Not sure) and knowledge of health insurance covering dental care (Yes, No, Not sure).

#### 2.4.2 Bivariate analysis for the whole household

Bivariate analysis was conducted using Chi-square to explore the relationships between demographics and dental care access variables among all participants. Demographic variables included country of origin (Afghanistan, Burma, DRC, Nepal, Somalia, and Syria) and years in the US (<1 year, 1–<5 years, 5–<10 years, and 10+ years). Dental access variables included the presence of a dentist in home countries (Yes, No, Not sure, Not applicable), the presence of a dentist in the US (Yes, No, Not sure, Not applicable), knowing where to receive dental care in the home country (dentist, primary care physician, urgent care, emergent care, other), and knowing where to receive dental care in the US (dentist, primary care physician, urgent care, emergent care, other).

### 2.5 Sharing findings with the community

The findings will be shared with CHWs who assisted with data collection by distributing fact sheets summarizing the results. The authors are finalizing the summary sheet as of September 2025. Our project team members also meet with most of the CHWs on a weekly basis, allowing for regular discussions about the survey results. The team has ongoing projects collaborating with the CHWs and will incorporate findings into future oral health education and outreach activities.

### 2.6 Ethics and consent

All participants or their legal guardians provided verbal informed consent to participate in the study and for their data to be published. The study was reviewed and deemed exempt from human subjects regulations by the SUNY Upstate Medical University Institutional Review Board (Project #2037903-2).

## 3. Results

### 3.1 Demographic characteristics

A total of 60 household, including 313 family members, participated in the study. Table 1 shows demographic characteristics for heads of household and individuals surveyed. Most heads of household were male (75%), and most individuals surveyed were female (51.8%). Of the categories listed, most heads of household reported being 30–<40 years old (28.3%). The most reported languages were Arabic (16.7%) and Nepali (16.7%) among heads of household and Arabic (22.0%) among individuals. Household most commonly had 1–5 family members (53.3%). Over half of the heads of household (51.7%) and just under half of individuals (46.0%) indicated they had lived in the US for 1–<5 years. See Table 1 for more details.

**Table 1:** Demographic characteristics among heads of household and individuals.

CHARACTERISTIC	*N* (%) HEAD OF HOUSEHOLD, *N* = 60	*N* (%) INDIVIDUAL, *N* = 313
**Demographics**		
Sex		
Male	45 (75.0)	151 (48.2)
Female	15 (25.0)	162 (51.8)
Age		
<5 years	—	31 (9.9)
5–<10 years	—	48 (15.3)
10–<20 years	—	92 (29.4)
20–<30 years	—	45 (14.4)
<30 years	8 (13.3)	—
30–<40 years	17 (28.3)	39 (12.5)
40–<50 years	14 (23.3)	30 (9.6)
50–<60 years	12 (20.0)	16 (5.1)
>60 years	9 (15.0)	—
60–<70 years	—	6 (1.9)
>70	—	6 (1.9)
Country of origin		
Afghanistan	10 (16.7)	51 (16.3)
Bhutan/Nepal	10 (16.7)	55 (17.6)
Burma	10 (16.7)	45 (14.4)
DRC	10 (16.7)	69 (22.0)
Somalia	10 (16.7)	24 (7.7)
Syria	10 (16.7)	69 (22.0)
Primary language		
Arabic	10 (16.7)	69 (22.0)
Dari	4 (6.7)	14 (4.5)
Karen	7 (11.7)	27 (8.6)
Karenni	2 (3.3)	13 (4.2)
Nepali	10 (16.7)	55 (17.6)
Pashto	6 (10.0)	37 (11.8)
Somali	8 (13.3)	22 (7.0)
Swahili	9 (15.0)	65 (20.8)
Missing	4 (6.7)	11 (3.5)
Number of family members in home		
One to five	32 (53.3)	—
Six to ten	26 (43.3)	—
More than 10	2 (3.3)	—
How long have you been in the US?		
<1 year	—	15 (4.8)
1–<5 years	31 (51.7)	144 (46.0)
5–<10 years	14 (23.3)	69 (22.0)
10+ years	15 (25.0)	48 (15.3)
Missing	0 (0.0)	37 (11.8)
Do you have any health insurance?		
No	3 (5.0)	3 (1.0)
Yes	57 (95.0)	310 (99.0)

### 3.2 Descriptive analysis of oral health literacy and access barriers

Table 2 shows most heads of household (60.0%) and individuals (56.2%) reported knowing their insurance covered dental care. Heads of household reported the three most common barriers in Syracuse, NY, to accessing dental care were language (53.3%), ability to find a dentist (48.3%), and insurance (35.0%). Almost half of the individuals surveyed did not have a dentist in their home country (47.9%), and had a dentist in the US (48.5%). Of the categories listed, slightly more than half of individuals indicated they saw a dentist when experiencing a dental problem in the US (51.4%), and over half reported not having a current dental problem (56.9%). See Table 2 for more details.

**Table 2:** Insurance knowledge, health behaviors, attitudes toward dental care, and challenges to seeking dental care for heads of household and individuals.

CHARACTERISTIC	*N* (%) HEAD OF HOUSEHOLD	*N* (%) INDIVIDUAL
**Insurance knowledge**		
Do you know if your health insurance covers dental care?		
No	5 (8.3)	31 (9.9)
Yes	36 (60.0)	176 (56.2)
Missing	19 (31.7)	106 (33.9)
Do you know the clinics that accept your insurance?		
No	13 (21.7)	67 (21.4)
Yes	29 (48.3)	145 (46.3)
Missing	18 (30.0)	101 (32.3)
**Health behaviors**		
How many times do you brush your teeth in a day?		
Once in a couple of days	2 (3.3)	—
One time per day	16 (26.7)	—
Two times per day	38 (63.3)	—
More than two times per day	4 (6.7)	—
Do you use floss?		
No	41 (68.3)	—
Yes	19 (31.7)	—
How often do you drink tap water?		
Everyday	22 (36.7)	—
Never	18 (30.0)	—
Not every day, but sometimes	20 (33.3)	—
When was your last visit to the dentist?		
Less than 1 year ago	16 (26.7)	—
1–<2 years ago	13 (21.7)	—
2–<5 years ago	8 (13.3)	—
>5 years ago	10 (16.7)	—
Never	6 (10.0)	—
Unsure	7 (11.7)	—
**Attitude toward dental care**		
Please rate how you feel about the importance of dental care on a scale of 1–5		
1	0 (0.0)	—
2	3 (5.0)	—
3	9 (15.0)	—
4	8 (13.3)	—
5	40 (66.7)	—
Have you ever planned to go abroad to receive dental care?		
No	43 (71.7)	—
Yes	7 (11.7)	—
I am planning to	10 (16.7)	—
How would you describe your oral health?		
Excellent	12 (20.0)	—
Good	20 (33.3)	—
Fair	19 (31.7)	—
Poor	9 (15.0)	—
At what stage of teething do you start your child’s dental care?		
At the start of teething	14 (23.3)	—
When all the teeth are in	17 (28.3)	—
When the child complains of pain	12 (20.0)	—
Not sure	17 (28.3)	—
**Challenges to seeking dental care**		
What are the challenges to seeking dental care? (Select all that apply)		
Transportation	13 (21.7)	—
Language	32 (53.3)	—
Time	19 (31.7)	—
Insurance	21 (35.0)	—
Ability to find a dentist	29 (48.3)	—
Financial	4 (6.7)	—
Not valuing dental care	2 (3.3)	—
Anxiety about a new place	1 (1.7)	—
Anxiety about dental care	9 (15.0)	—
Do you have a dentist in your home country?		
No	—	150 (47.9)
Yes	—	75 (24.0)
Missing	—	88 (28.1)
Do you have a dentist in the US?		
No	—	142 (45.4)
Yes	—	152 (48.5)
Missing	—	19 (6.1)
Who did you go to when you had a dental problem in your home country?		
Dentist	—	130 (41.5)
Emergent care	—	9 (2.9)
Primary care physician	—	60 (19.2)
Urgent care	—	14 (4.5)
Other	—	100 (31.9)
Who do you go to when you have a dental problem in the US?		
Dentist	—	161 (51.4)
Emergent care	—	23 (7.3)
Primary care physician	—	34 (10.9)
Urgent care	—	37 (11.8)
Other	—	58 (18.5)
Do you have any dental problems now?		
No	—	178 (56.9)
Yes	—	124 (39.6)
Missing	—	11 (3.5)

### 3.3 Bivariate analysis for dental care access

Fisher’s exact tests revealed no association between country of origin and heads of household having a dentist in the US (*p* = 0.055). We also explored relationships between health literacy variables, including brushing habits, flossing habits, time of last visit to the dentist, what stage of teething parents think they should start their child’s dental care, and self-reported dental condition, with heads of household having a dentist in the US. Among these, only the time of the last dental visit was significantly associated with the presence of a US dentist for heads of household (*p* < 0.05).

An exploratory analysis also looked at associations between potential barriers to dental care, including country of origin and time in the US, with the US dental care access variables, having a dentist and actual location where all participants received dental care in the US such as dentist, emergent care, primary care physician, urgent care, and others.

For all participants, Chi-square results revealed a significant association between country of origin and having a dentist in the US (Supplemental Figure 1) and location of dental care in the US (Supplemental Figure 2). Years in the US were significantly associated with having a dentist among all participants (Supplemental Figure 3, *p* < 0.001) and among heads of household (Supplemental Figure 4, *p* < 0.001). Among all participants, the proportion reporting a dentist was relatively high among those with less than 1 year of residence. In the 1–<5 year group, however, only a small minority reported having a dentist compared with those who did not. Access improved after five years of residence: in both the 5–<10 year and 10+ year groups, more participants reported having a dentist than not. In particular, the 10+ year group showed the highest ratio, with the vast majority reporting a dentist (42 of 47). In contrast, heads of household showed a clearer pattern: the number of people reporting having a dentist was higher with longer residency. Additionally, the location of dental care was also significantly associated with years in the US for all participants (Supplemental Figure 5, *p* < 0.001) and heads of household (Supplemental Figure 6, *p* < 0.001). The use of primary care physicians for dental care increased among participants who had been in the US for more than 10 years.

## 4. Discussion

The findings from this study provide critical insights into dental care access and barriers faced by the refugee population in Syracuse, NY.

### 4.1 Current dental care access among refugees

In our descriptive study, even though only 24.0% of respondents had dental care access in their home country, 48.5% reported having dental care access in the US. This suggests an improvement in dental care accessibility upon resettlement. Nevertheless, access to dental care among the refugee population in the US is worse than that of adults and children in America, which is 65.5% and 86.9%, respectively, as shown by the data of the Centers for Disease Control and Prevention (CDC) [31]. Only 26.7% of heads of household in this study reported having visited a dentist within one year, which is significantly lower than rates published in similar refugee populations living in Massachusetts (54%) [13] and Texas (37%) [2]. The American Dental Association (ADA) recommends seeing dentists regularly, twice a year, for preventive care and early treatment [32]. Expanding dental facilities that can accommodate the refugee population and improving health literacy to encourage refugee people to visit dentists are important for their oral health care.

### 4.2 Seeking dental care abroad

Several studies have reported that one-third of immigrants in the US have obtained dental care abroad [33, 34]. Similarly, our data revealed that nearly 30% of refugee heads of household (28.4%)—combining those who responded “yes” and those who responded “I am planning to”—reported seeking or planning to seek dental care outside the US due to poor dental care access in the US. This is an increasing trend as reports have shown that many Americans are seeking alternative, affordable dental care options due to the rising cost of dental care in the US [35]. A population-based surveillance study found hundreds of thousands of US residents traveled abroad for dental or medical procedures annually [34]. While this number is significant, no specific data have been available on the number of US refugees seeking dental care abroad due to high costs or limited access. Our study documented that a growing trend of going abroad for dental care was also found among refugee populations in the US. This suggests that targeted policy interventions to improve dental care access and affordability for vulnerable populations should be considered.

### 4.3 Self-reported oral condition

Among the heads of household, 46.7% rated their oral condition as fair or poor, and 39.6% of all respondents reported having dental problems. This self-reported oral condition may have been underestimated, given their oral health literacy. A previous study of refugees in San Antonio, Texas, showed that 47.3% of respondents evaluated their oral condition as poor or very poor [2]. The similarity between the self-reported oral conditions in our study and the findings from Antonio, Texas, suggests that poor oral health may be a widespread issue among refugee populations in the US. In a broader study comparing American citizens and non-citizens, 26.2% of American citizens rated their oral health as fair or bad [36], which is lower than reported percentages in the refugee populations. This disparity highlights oral health challenges faced by refugees compared to the general population.

### 4.4 Oral health literacy and behavior

The questions about respondents’ current oral health behaviors demonstrated that 63.3% of respondents reported brushing their teeth twice a day and 31.7% used floss. A previous survey in Massachusetts noted that 74.5% reported brushing at least twice a day, 40% reported flossing [13], and another study in Texas showed that 43% brushed twice a day, and 17.4% used dental floss. Data targeting general American residents showed that about 70% of American people brush their teeth twice a day [37], which is consistent with or slightly higher than the percentage of the refugee population. For flossing, one study using American national data demonstrated that about 30% of American adults floss daily [38], which was comparable to the number in the refugee population.

Furthermore, our study found that only 23.3% knew that children’s dental care should be started just after teething. Current guidance states that brushing teeth should be started as soon as the first teeth emerge [39]. A previous study targeting Somali refugees in Massachusetts showed that participants with higher health literacy practiced more preventive care [13]. It is important to provide oral health education, including care for children.

The association between having a US dentist and the timing of the last dental visit highlights the importance of dental education and literacy. Although flossing habits showed a trend toward significance (*p* = 0.057), the association did not reach statistical significance. Nevertheless, improving oral health literacy among refugees remains crucial, as it may encourage greater engagement with dental services and promote better oral hygiene practices. Guaranteeing refugees’ oral healthcare access will improve not only their oral condition but also their health literacy, which will boost their preventive care behavior.

### 4.5 Barriers to dental care

The three most reported barriers to dental care were language (53.3%), difficulty finding a dentist (48.3%), and insurance issues (35.0%). These barriers are consistent with known challenges that refugees face in accessing healthcare services [2, 7–11], emphasizing the need for targeted interventions to address these issues across both the healthcare and dental care sectors. The barrier caused by differences in insurance coverage has been reported in previous studies [4, 5, 10], and despite ongoing efforts, it continues to persist. Continuous dialog and intervention at the system level are needed.

### 4.6 Years of stay in the US and dental care access and location

Our analysis indicated that dental access varied according to length of residence in the US, with different patterns observed for heads of household and all participants. Among all participants, only 10 people reported having a dentist in the 1–<5 year residency group, compared with 47 people in the 5–<10 year group and 42 people in the 10+ year group, indicating that dental access improved after 5 years of residency. This suggests that the early years after resettlement represent a particularly vulnerable period for dental access, but that longer term residence can eventually facilitate stable connections with dental providers. For heads of household, the pattern was similar, with limited access in the first 5 years but a greater number of people reporting having a dentist after longer residence. These findings partly align with a previous study reporting that those who had resettled more recently to the US were less likely to have seen a dentist in the past 5 years and less likely to see a dentist for regular checkups or consultations, indicating that time in the US may contribute to improved dental access [2].

Furthermore, our finding that reliance on primary care physicians for dental needs increased among those with more than 10 years of residence underscores that long-term presence in the US does not eliminate systemic barriers. Together, these results emphasize the need for interventions that address both the immediate challenges faced by newly arrived refugees and the sustained, family-wide barriers that persist over time.

### 4.7 Country of origin and dental care access

Although the association between country of origin and having a dentist in the US did not reach statistical significance when tested with Fisher’s exact test (*p* = 0.055), Chi-square results suggested meaningful differences across each group of country of origin, reflecting the diverse experiences and challenges faced by different refugee groups. For example, participants from Bhutan and Burma were more likely to report having a dentist, while those from Syria and Somalia showed roughly equal proportions of having and not having a dentist. In contrast, Afghan participants were more likely not to have a dentist, despite their documented high dental needs related to nutritional deprivation and inadequate living conditions prior to resettlement [40]. Such variations may be based on cultural differences, language spoken, healthcare system, and access in their countries of origin, as described in previous studies [4, 8]. These findings underscore the necessity for culturally sensitive and tailored oral healthcare approaches, considering the heterogeneity of refugee populations.

### 4.8 New contribution to the literature

This study was the first dental care and oral health survey targeting newly resettled refugees in the US from various countries of origin. The survey results point to several policy implications. First, enhancing language services and interpreter availability could mitigate one of the primary barriers to dental care. Second, increasing awareness and simplifying the process of finding dental providers for refugees could improve access rates. Lastly, addressing insurance-related barriers through policy adjustments or targeted programs could ensure better coverage and affordability of dental care for refugee populations.

### 4.9 Limitations

Despite these strengths, this study has several limitations. First, selection bias is possible because participants were selected through snowball sampling of CHWs. However, this participant recruitment was proper, considering the necessity of translation by CHWs and smooth recruitment by trusted people who are CHWs in the refugee population. Second, it is acknowledged that this study may have been subjected to recall bias. Information about all household members was obtained from the head of the household. To mitigate this bias, CHWs who interviewed respondents made efforts to clearly explain the questions to respondents, and they were encouraged to consider each family member carefully when responding. However, the possibility of recall errors remains, and future studies collecting data directly from each household member could be considered. Third, interviewing bias could affect this survey. The present survey inquired about refugee populations through interpretations by CHWs. Therefore, there is a possibility that interpretation could change the meaning of the survey questions and responses. However, researchers and CHWs communicated extensively about the research aims and the implications of the survey questions. Thus, researchers believe that the impact of interviewing bias was minimized. Fourth, the questions used in the present survey were created by researchers and have not been validated by previous studies. Nevertheless, all questions were discussed based on community outreach and project members who have worked with the refugee population in the community and clinic. Fifth, the educational level was missing in the demographic questions, which might strongly affect oral health literacy. A future study including this variable is needed for a more detailed analysis. Sixth, the multivariate analysis was not conducted in this study due to statistical error caused by the lack of responses for some questions. However, the descriptive study and bivariate analysis provided meaningful indications. A seventh limitation concerns the cross-sectional nature of this study. Cross-sectional studies limit generalizability to other populations. An eighth limitation is that the relationship between participants and their CHWs may have influenced responses. Familiarity with CHWs likely enhanced trust and facilitated more open communication. Still, it may also have introduced bias, for example, by shaping participants’ willingness to disclose sensitive barriers to dental care. We consider it more likely that the CHWs’ established roles as trusted community members strengthened, rather than undermined, the reliability of the data, yet this remains a potential source of bias. A ninth limitation is that self-reported oral health behaviors, such as tooth brushing and flossing, may not reflect whether these actions were performed correctly. While this limits the strength of conclusions drawn from these items, the trust participants placed in CHWs likely promoted honest disclosure, supporting the credibility of the frequency data.

Lastly, because the survey was administered in multiple languages with real-time translation by CHWs, some cultural and linguistic nuances may not have been fully preserved. To address this, the research team and CHWs engaged in ongoing discussions throughout the study to share contextual understanding of refugees’ limited access to dental care in the United States, which helped promote consistency in interpretation across translations.

## 5. Conclusions

This study illuminates the significant barriers to dental care faced by refugee households in Syracuse, NY, and highlights the need for comprehensive strategies to address these challenges. Improving dental care access and reducing barriers can enhance refugee populations’ overall health and well-being, fostering their successful integration into the US healthcare system. Future research should focus on longitudinal studies to monitor changes over time and evaluate the effectiveness of implemented interventions.

## Figures and Tables

**Table:** 

US	United States
NY	New York
NYS	New York State
SUNY	State University of New York
CYO	Catholic Charities Refugee Resettlement Services
CCP	Community-Clinic Partnership
CHWs	Community Health Workers
IRB	Institutional Review Board
DRC	Democratic Republic of the Congo
CDC	Centers for Disease Control and Prevention
ADA	American Dental Association

## Data Availability

The data that support the findings of this study are available on request from the corresponding author. The data are not publicly available due to privacy or ethical restrictions.

## Additional Files

The additional files for this article can be found as follows:

Supplementary Figure 1. [X^2^(10) = 94.62, *p* < 0.001].

Supplementary Figure 2. [X^2^(20) = 266.51, *p* < 0.001].

Supplementary Figure 3. [X^2^(3) = 74.88, *p* < 0.001].

Supplementary Figure 4. [X^2^(2) = 15.87, *p* < 0.001].

Supplementary Figure 5. [X^2^(12) = 65.01, *p* < 0.001].

Supplementary Figure 6. [X^2^(12) = 65.01, *p* < 0.001].

Supplementary Data. Refugees Dental Access Survey.
