# Supplementary figures and images for: Oral Health and Dental Care Access Among Refugees in Syracuse, NY
Source: Ann Glob Health. 2025 Nov 3;91(1):76. doi: 10.5334/aogh.4739 (PMC12593417; doi:10.5334/aogh.4739)

## Supplementary Figure 1

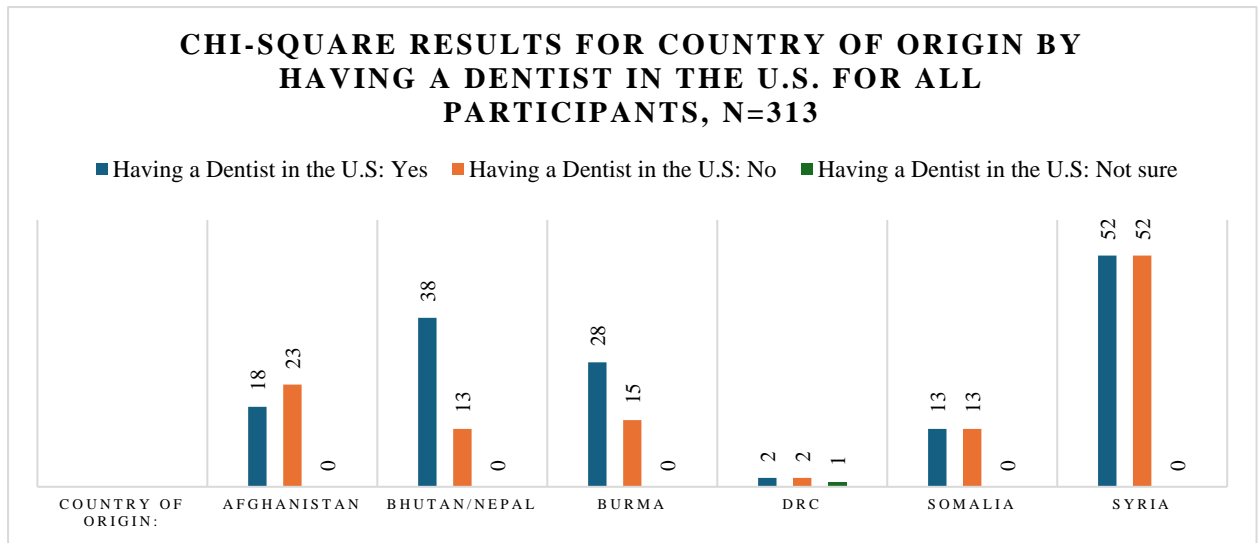

Figure 1: [ $\chi^2(10)=94.62, p<0.001$ ]

Supplement: Supplementary Figure 1. — [X2(10) = 94.62, p < 0.001]. [file agh-91-1-4739-s1.pdf]

## Supplementary Figure 2

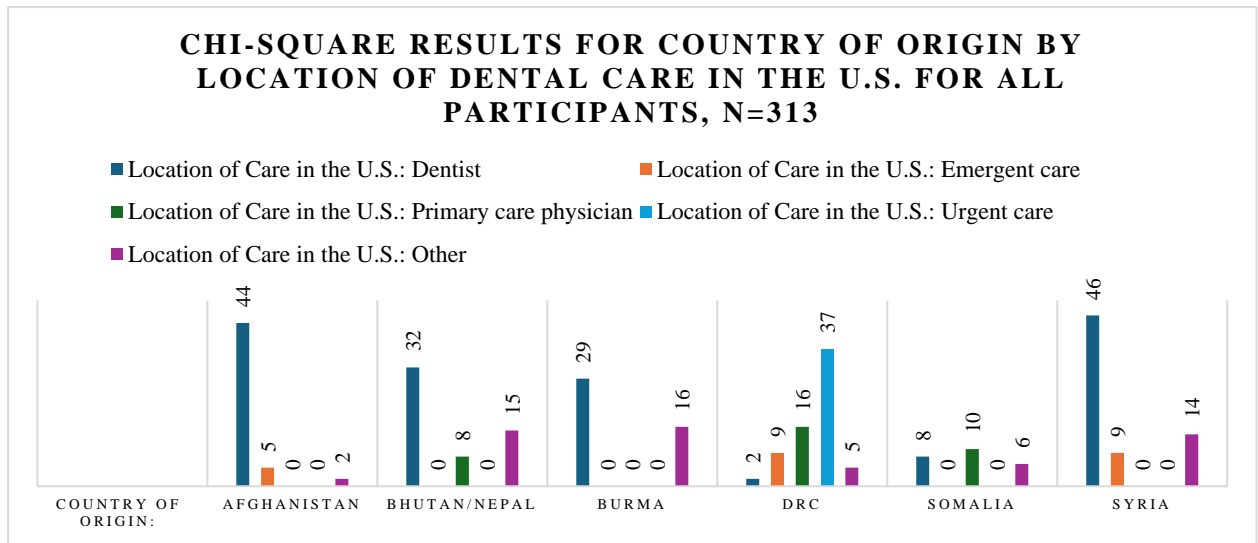

Figure 2: [ $X^2(20)=266.51, p<0.001$ ]

Supplement: Supplementary Figure 2. — [X2(20) = 266.51, p < 0.001]. [file agh-91-1-4739-s2.pdf]

### Supplementary Figure 3

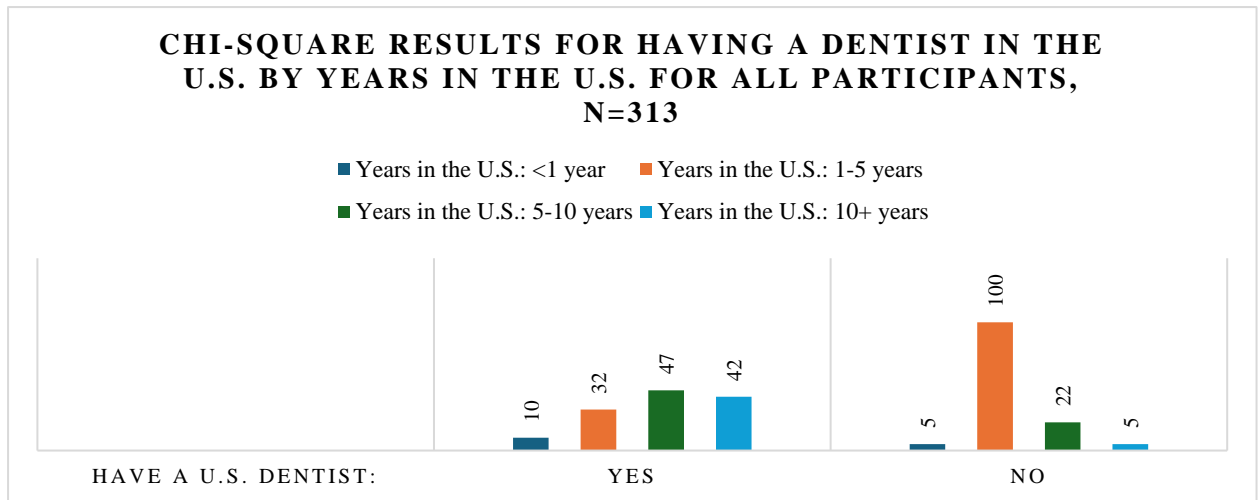

Figure 3: [ $X^2(3)=74.88$ ,  $p<0.001$ ]

Supplement: Supplementary Figure 3. — [X2(3) = 74.88, p < 0.001]. [file agh-91-1-4739-s3.pdf]

## Supplementary Figure 4

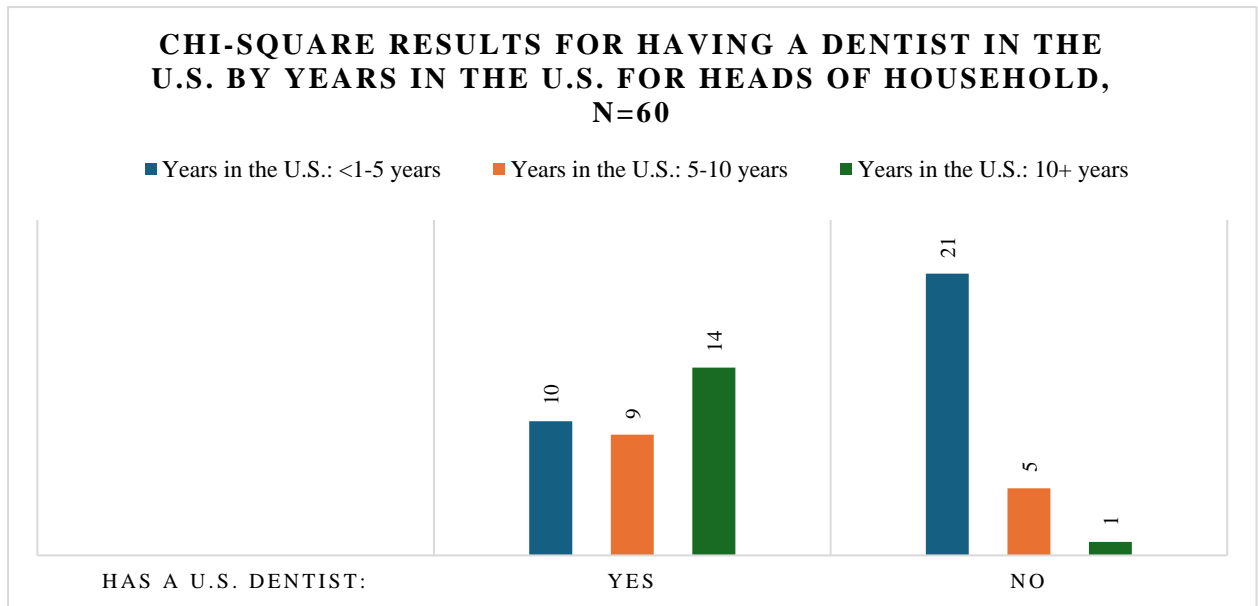

Figure 4: [ $X^2(2)=15.87$ ,  $p<0.001$ ]

Supplement: Supplementary Figure 4. — [X2(2) = 15.87, p < 0.001]. [file agh-91-1-4739-s4.pdf]

Supplementary Figure 5

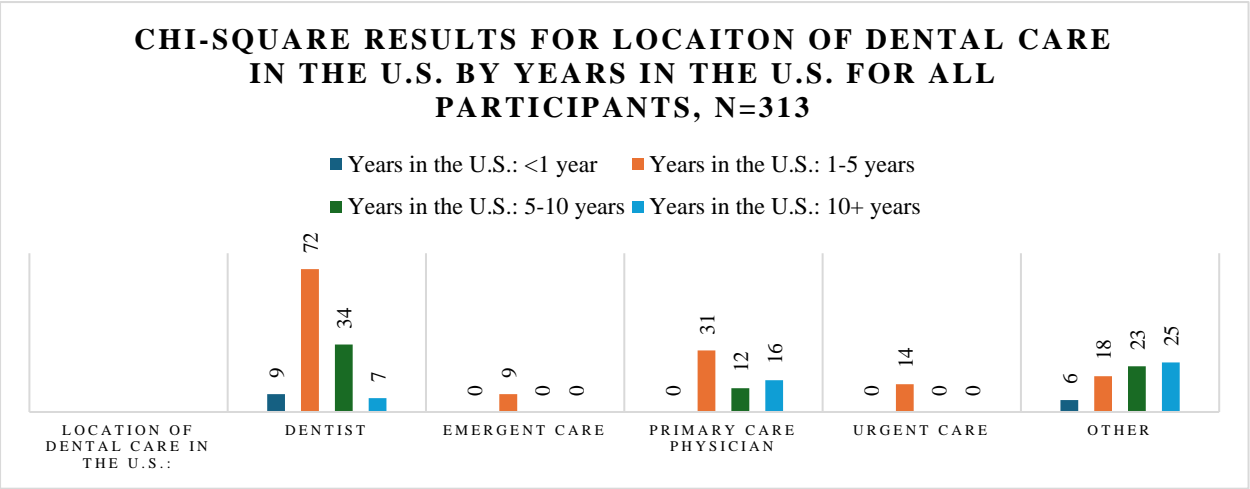

Figure 5: [ $X^2(12)=65.01, p<0.0$ ]

Supplement: Supplementary Figure 5. — [X2(12) = 65.01, p < 0.001]. [file agh-91-1-4739-s5.pdf]

## Supplementary Figure 6

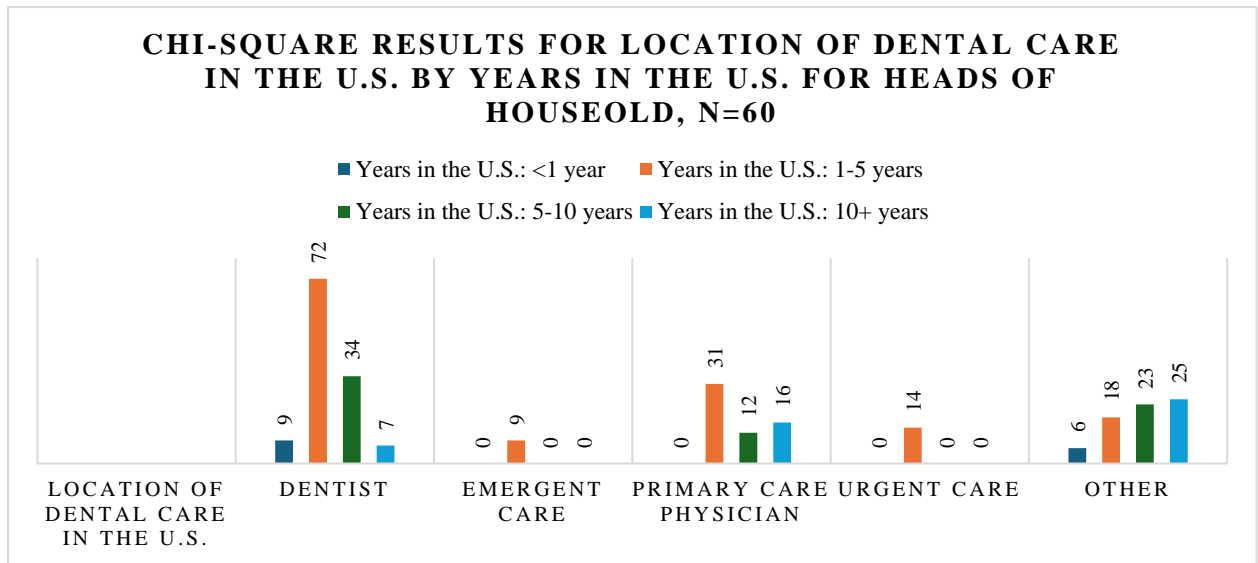

Figure 6: [ $X^2(12)=65.01$ ,  $p<0.001$ ]

Supplement: Supplementary Figure 6. — [X2(12) = 65.01, p < 0.001]. [file agh-91-1-4739-s6.pdf]
