# Supplementary material for: Oral Health and Dental Care Access Among Refugees in Syracuse, NY
Source: Ann Glob Health. 2025 Nov 3;91(1):76. doi: 10.5334/aogh.4739 (PMC12593417; doi:10.5334/aogh.4739)
Supplement: Supplementary Data. — Refugees Dental Access Survey. [file agh-91-1-4739-s7.pdf]

Refugees Dental Access Survey

Please complete the survey below.

This survey aims to know the situation of oral health and dental care among the refugee population in Syracuse. It will take 10 to 15 minutes to answer.

All answers will be collected anonymously and used only for analysis and publication related to a quality improvement project for refugee dental care in Syracuse by Community-Clinical Partnership.

Thank you in advance for completing this important survey.

[To Refugee Health Navigators]

Please ask ALL questions to refugee families and input answers in the survey below.

-Community-Clinic Partnership team-

0 Are you a health navigator or case manager?
- ◯ Yes
- ◯ No (I am a refugee client)

Part 1. Demographics

1 What is your country of origin? ______________________________

2 What is your primary language? ______________________________

3 How long have you been in the U.S.? (Please write down XX years) ______________________________

4 Do you have any health insurance?
- ◯ Yes
- ◯ No

5 Do you know if your health insurance covers dental care?
- ◯ Yes
- ◯ No
- ◯ Not sure

6 Do you know the clinics accept your insurance?
- ◯ Yes
- ◯ No
- ◯ Not sure

Part 2. Family information

Please list your family members IN ORDER OF AGE (answer from the oldest member), including YOU.
If you answer "yes" to a question "you have one or more family members" at the end of this section, more questions about other family members will be displayed.

7 How many family members are in your home? ______________________________

8-1a Please answer your family member's age. (Family member 1) *Please start with the oldest member. ______________________________

8-1b	Please answer your family member's gender. (Family member 1)	◯ Male<br>◯ Female<br>◯ Other<br>◯ Not prefer to answer
8-1c	Does your family member 1 have a dentist at home country?	◯ Yes<br>◯ No<br>◯ Not sure<br>◯ Not Applicable
8-1d	Does your family member 1 have a dentist in the United States?	◯ Yes<br>◯ No<br>◯ Not sure<br>◯ Not Applicable
8-1e	Who did your family member 1 go to when he/she has a dental problem in your home country?	◯ Dentist<br>◯ Primary care physician<br>◯ Urgent care<br>◯ Emergent care<br>◯ Other
	Please describe it.	__________________________________
8-1f	Who does your family member 1 go to when he/she has a dental problem in the United States?	◯ Dentist<br>◯ Primary care physician<br>◯ Urgent care<br>◯ Emergent care<br>◯ Other
	Please describe it.	__________________________________
8-1g	Does your family member 1 have any dental problems now?	◯ Yes<br>◯ No<br>◯ Not sure
8-1h	You have one or more family members.<br>(If you click "Yes", more questions about the next family member will be displayed.)	◯ Yes<br>◯ No
8-2a	Please answer your family member's age. (Family member 2)	__________________________________
8-2b	Please answer your family member's gender. (Family member 2)	◯ Male<br>◯ Female<br>◯ Other<br>◯ Not prefer to answer
8-2c	Does your family member 2 have a dentist at home country?	◯ Yes<br>◯ No<br>◯ Not sure<br>◯ Not Applicable
8-2d	Does your family member 2 have a dentist in the United States?	◯ Yes<br>◯ No<br>◯ Not sure<br>◯ Not Applicable

8-2e Who did your family member 2 go to when he/she has a dental problem in your home country?

◯ Dentist
◯ Primary care physician
◯ Urgent care
◯ Emergent care
◯ Other

Please describe it.

____

8-2f Who does your family member 2 go to when he/she has a dental problem in the United States?

◯ Dentist
◯ Primary care physician
◯ Urgent care
◯ Emergent care
◯ Other

Please describe it.

____

8-2g Does your family member 2 have any dental problems now?

◯ Yes
◯ No
◯ Not sure

8-2h You have one or more family members.

◯ Yes
◯ No

8-3a Please answer your family member's age. (Family member 3)

____

8-3b Please answer your family member's gender. (Family member 3)

◯ Male
◯ Female
◯ Other
◯ Not prefer to answer

8-3c Does your family member 3 have a dentist at home country?

◯ Yes
◯ No
◯ Not sure
◯ Not Applicable

8-3d Does your family member 3 have a dentist in the United States?

◯ Yes
◯ No
◯ Not sure
◯ Not Applicable

8-3e Who did your family member 3 go to when he/she has a dental problem in your home country?

◯ Dentist
◯ Primary care physician
◯ Urgent care
◯ Emergent care
◯ Other

Please describe it.

____

8-3f Who does your family member 3 go to when he/she has a dental problem in the United States?

◯ Dentist
◯ Primary care physician
◯ Urgent care
◯ Emergent care
◯ Other

Please describe it. __________________________________

8-3g Does your family member 3 have any dental problems now?
- ◯ Yes
- ◯ No
- ◯ Not sure

8-3h You have one or more family members.
- ◯ Yes
- ◯ No

8-4a Please answer your family member's age. (Family member 4) __________________________________

8-4b Please answer your family member's gender. (Family member 4)
- ◯ Male
- ◯ Female
- ◯ Other
- ◯ Not prefer to answer

8-4c Does your family member 4 have a dentist at home country?
- ◯ Yes
- ◯ No
- ◯ Not sure
- ◯ Not Applicable

8-4d Does your family member 4 have a dentist in the United States?
- ◯ Yes
- ◯ No
- ◯ Not sure
- ◯ Not Applicable

8-4f Who does your family member 4 go to when he/she has a dental problem in the United States?
- ◯ Dentist
- ◯ Primary care physician
- ◯ Urgent care
- ◯ Emergent care
- ◯ Other

Please describe it. __________________________________

8-4e Who did your family member 4 go to when he/she has a dental problem in your home country?
- ◯ Dentist
- ◯ Primary care physician
- ◯ Urgent care
- ◯ Emergent care
- ◯ Other

Please describe it. __________________________________

8-4g Does your family member 4 have any dental problems now?
- ◯ Yes
- ◯ No
- ◯ Not sure

8-4h You have one or more family members.
- ◯ Yes
- ◯ No

8-5a Please answer your family member's age. (Family member 5) __________________________________

8-5b Please answer your family member's gender. (Family member 5)

◯ Male
◯ Female
◯ Other
◯ Not prefer to answer

8-5c Does your family member 5 have a dentist at home country?

◯ Yes
◯ No
◯ Not sure
◯ Not Applicable

8-5d Does your family member 5 have a dentist in the United States?

◯ Yes
◯ No
◯ Not sure
◯ Not Applicable

8-5e Who did your family member 5 go to when he/she has a dental problem in your home country?

◯ Dentist
◯ Primary care physician
◯ Urgent care
◯ Emergent care
◯ Other

Please describe it.

____

8-5f Who does your family member 5 go to when he/she has a dental problem in the United States?

◯ Dentist
◯ Primary care physician
◯ Urgent care
◯ Emergent care
◯ Other

Please describe it.

____

8-5g Does your family member 5 have any dental problems now?

◯ Yes
◯ No
◯ Not sure

8-5h You have one or more family members.

◯ Yes
◯ No

8-6a Please answer your family member's age. (Family member 6)

____

8-6b Please answer your family member's gender. (Family member 6)

◯ Male
◯ Female
◯ Other
◯ Not prefer to answer

8-6c Does your family member 6 have a dentist at home country?

◯ Yes
◯ No
◯ Not sure
◯ Not Applicable

8-6d Does your family member 6 have a dentist in the United States?

◯ Yes
◯ No
◯ Not sure
◯ Not Applicable

8-6e Who did your family member 6 go to when he/she has a dental problem in your home country?

- ◯ Dentist
- ◯ Primary care physician
- ◯ Urgent care
- ◯ Emergent care
- ◯ Other

Please describe it.

____

8-6f Who does your family member 6 go to when he/she has a dental problem in the United States?

- ◯ Dentist
- ◯ Primary care physician
- ◯ Urgent care
- ◯ Emergent care
- ◯ Other

Please describe it.

____

8-6g Does your family member 6 have any dental problems now?

- ◯ Yes
- ◯ No
- ◯ Not sure

8-6h You have one or more family members.

- ◯ Yes
- ◯ No

8-7a Please answer your family member's age. (Family member 7)

____

8-7b Please answer your family member's gender. (Family member 7)

- ◯ Male
- ◯ Female
- ◯ Other
- ◯ Not prefer to answer

8-7c Does your family member 7 have a dentist at home country?

- ◯ Yes
- ◯ No
- ◯ Not sure
- ◯ Not Applicable

8-7d Does your family member 7 have a dentist in the United States?

- ◯ Yes
- ◯ No
- ◯ Not sure
- ◯ Not Applicable

8-7e Who did your family member 7 go to when he/she has a dental problem in your home country?

- ◯ Dentist
- ◯ Primary care physician
- ◯ Urgent care
- ◯ Emergent care
- ◯ Other

Please describe it.

____

8-7f Who does your family member 7 go to when he/she has a dental problem in the United States?

- ◯ Dentist
- ◯ Primary care physician
- ◯ Urgent care
- ◯ Emergent care
- ◯ Other

Please describe it. ______________________

8-7g Does your family member 7 have any dental problems now?
- ◯ Yes
- ◯ No
- ◯ Not sure

8-7h You have one or more family members.
- ◯ Yes
- ◯ No

8-8a Please answer your family member's age. (Family member 8) ______________________

8-8b Please answer your family member's gender. (Family member 8)
- ◯ Male
- ◯ Female
- ◯ Other
- ◯ Not prefer to answer

8-8c Does your family member 8 have a dentist at home country?
- ◯ Yes
- ◯ No
- ◯ Not sure
- ◯ Not Applicable

8-8d Does your family member 8 have a dentist in the United States?
- ◯ Yes
- ◯ No
- ◯ Not sure
- ◯ Not Applicable

8-8e Who did your family member 8 go to when he/she has a dental problem in your home country?
- ◯ Dentist
- ◯ Primary care physician
- ◯ Urgent care
- ◯ Emergent care
- ◯ Other

Please describe it. ______________________

8-8f Who does your family member 8 go to when he/she has a dental problem in the United States?
- ◯ Dentist
- ◯ Primary care physician
- ◯ Urgent care
- ◯ Emergent care
- ◯ Other

Please describe it. ______________________

8-8g Does your family member 8 have any dental problems now?
- ◯ Yes
- ◯ No
- ◯ Not sure

8-8h You have one or more family members.
- ◯ Yes
- ◯ No

8-9a Please answer your family member's age. (Family member 9) ______________________

8-9b Please answer your family member's gender. (Family member 9)
- ◯ Male
- ◯ Female
- ◯ Other
- ◯ Not prefer to answer

8-9c Does your family member 9 have a dentist at home country?
- ◯ Yes
- ◯ No
- ◯ Not sure
- ◯ Not Applicable

8-9d Does your family member 9 have a dentist in the United States?
- ◯ Yes
- ◯ No
- ◯ Not sure
- ◯ Not Applicable

8-9e Who did your family member 9 go to when he/she has a dental problem in your home country?
- ◯ Dentist
- ◯ Primary care physician
- ◯ Urgent care
- ◯ Emergent care
- ◯ Other

Please describe it. ______________________________

8-9f Who does your family member 9 go to when he/she has a dental problem in the United States?
- ◯ Dentist
- ◯ Primary care physician
- ◯ Urgent care
- ◯ Emergent care
- ◯ Other

Please describe it. ______________________________

8-9g Does your family member 9 have any dental problems now?
- ◯ Yes
- ◯ No
- ◯ Not sure

8-9h You have one or more family members.
- ◯ Yes
- ◯ No

8-10a Please answer your family member's age. (Family member 10) ______________________________

8-10b Please answer your family member's gender. (Family member 10)
- ◯ Male
- ◯ Female
- ◯ Other
- ◯ Not prefer to answer

8-10c Does your family member 10 have a dentist at home country?
- ◯ Yes
- ◯ No
- ◯ Not sure
- ◯ Not Applicable

8-10d Does your family member 10 have a dentist in the United States?
- ◯ Yes
- ◯ No
- ◯ Not sure
- ◯ Not Applicable

8-10e Who did your family member 10 go to when he/she has a dental problem in your home country?

◯ Dentist
◯ Primary care physician
◯ Urgent care
◯ Emergent care
◯ Other

Please describe it.

____

8-10f Who does your family member 10 go to when he/she has a dental problem in the United States?

◯ Dentist
◯ Primary care physician
◯ Urgent care
◯ Emergent care
◯ Other

Please describe it.

____

8-10g Does your family member 10 have any dental problems now?

◯ Yes
◯ No
◯ Not sure

8-10h You have one or more family members.

◯ Yes
◯ No

8-11a Please answer your family member's age. (Family member 11)

____

8-11b Please answer your family member's gender. (Family member 11)

◯ Male
◯ Female
◯ Other
◯ Not prefer to answer

8-11c Does your family member 11 have a dentist at home country?

◯ Yes
◯ No
◯ Not sure
◯ Not Applicable

8-11d Does your family member 11 have a dentist in the United States?

◯ Yes
◯ No
◯ Not sure
◯ Not Applicable

8-11e Who did your family member 11 go to when he/she has a dental problem in your home country?

◯ Dentist
◯ Primary care physician
◯ Urgent care
◯ Emergent care
◯ Other

Please describe it.

____

8-11f Who does your family member 11 go to when he/she has a dental problem in the United States?

◯ Dentist
◯ Primary care physician
◯ Urgent care
◯ Emergent care
◯ Other

Please describe it. __________________________________

8-11g Does your family member 11 have any dental problems now?
- ◯ Yes
- ◯ No
- ◯ Not sure

8-11h You have one or more family members.
- ◯ Yes
- ◯ No

8-12a Please answer your family member's age. (Family member 12) __________________________________

8-12b Please answer your family member's gender. (Family member 12)
- ◯ Male
- ◯ Female
- ◯ Other
- ◯ Not prefer to answer

8-12c Does your family member 12 have a dentist at home country?
- ◯ Yes
- ◯ No
- ◯ Not sure
- ◯ Not Applicable

8-12d Does your family member 12 have a dentist in the United States?
- ◯ Yes
- ◯ No
- ◯ Not sure
- ◯ Not Applicable

8-12e Who did your family member 12 go to when he/she has a dental problem in your home country?
- ◯ Dentist
- ◯ Primary care physician
- ◯ Urgent care
- ◯ Emergent care
- ◯ Other

Please describe it. __________________________________

8-12f Who does your family member 12 go to when he/she has a dental problem in the United States?
- ◯ Dentist
- ◯ Primary care physician
- ◯ Urgent care
- ◯ Emergent care
- ◯ Other

Please describe it. __________________________________

8-12g Does your family member 12 have any dental problems now?
- ◯ Yes
- ◯ No
- ◯ Not sure

8-12h You have one or more family members.
- ◯ Yes
- ◯ No

8-13a Please answer your family member's age. (Family member 13) __________________________________

8-13b Please answer your family member's gender. (Family member 13)
- ◯ Male
- ◯ Female
- ◯ Other
- ◯ Not prefer to answer

8-13c Does your family member 13 have a dentist at home country?
- ◯ Yes
- ◯ No
- ◯ Not sure
- ◯ Not Applicable

8-13d Does your family member 13 have a dentist in the United States?
- ◯ Yes
- ◯ No
- ◯ Not sure
- ◯ Not Applicable

8-13e Who did your family member 13 go to when he/she has a dental problem in your home country?
- ◯ Dentist
- ◯ Primary care physician
- ◯ Urgent care
- ◯ Emergent care
- ◯ Other

Please describe it. ________________________________

8-13f Who does your family member 13 go to when he/she has a dental problem in the United States?
- ◯ Dentist
- ◯ Primary care physician
- ◯ Urgent care
- ◯ Emergent care
- ◯ Other

Please describe it. ________________________________

8-13g Does your family member 13 have any dental problems now?
- ◯ Yes
- ◯ No
- ◯ Not sure

8-13h If you have more family members, please write down the number of family members remained and move on to next part. ________________________________

Part 3. Perspectives on dental care

9 How many times do you brush your teeth a day?
- ◯ None
- ◯ Once a couple of days
- ◯ One time per a day
- ◯ Two times per a day
- ◯ More than two times per a day

10 Do you use floss?
- ◯ Yes
- ◯ No
- ◯ Not sure

11	When was your last visit to the dentist?	◯ Less than 12 months ago<br>◯ 1-2 years<br>◯ 2-5 years<br>◯ >5 years<br>◯ Never<br>◯ Unsure
12	At what stage of teething should you start your child's dental care?	◯ At the start of teething<br>◯ When all teeth are in<br>◯ When the child complains of pain<br>◯ Not sure
13	How would you describe your oral health?	◯ Excellent<br>◯ Good<br>◯ Fair<br>◯ Poor
14	What are the challenges to seeking dental care? (select ALL apply)	☐ Language<br>☐ Transportation<br>☐ Insurance<br>☐ Time<br>☐ Ability to find a dentist<br>☐ Financial<br>☐ Not valuing dental care<br>☐ Anxiety about a new place<br>☐ Anxiety about dental care
15	Please rate how you feel about the importance of dental care.	Not important — Fair — Very important<br>*(Place a mark on the scale above)*
16	Have you ever planned to go abroad to receive dental care?	◯ Yes ◯ No ◯ I am planning to
17	How often do you drink tap water?	◯ Everyday<br>◯ Not every day, but sometimes<br>◯ Never
